# Transposon insertion profiling by sequencing (TIPseq) for mapping LINE-1 insertions in the human genome

**DOI:** 10.1186/s13100-019-0148-5

**Published:** 2019-03-08

**Authors:** Jared P. Steranka, Zuojian Tang, Mark Grivainis, Cheng Ran Lisa Huang, Lindsay M. Payer, Fernanda O. R. Rego, Thiago Luiz Araujo Miller, Pedro A. F. Galante, Sitharam Ramaswami, Adriana Heguy, David Fenyö, Jef D. Boeke, Kathleen H. Burns

**Affiliations:** 1 Department of Pathology, Johns Hopkins University School of Medicine, Baltimore, MD 21205 USA; 2 McKusick–Nathans Institute of Genetic Medicine, Johns Hopkins University School of Medicine, Baltimore, MD 21205 USA; 3 Department for Biochemistry and Molecular Pharmacology, NYU Langone Health, New York, NY 10016 USA; 4 Institute for Systems Genetics, NYU Langone Health, New York, NY 10016 USA; 5 Centro de Oncologia Molecular, Hospital Sírio-Libanês, São Paulo, Brazil; 6 Departamento de Bioquímica, Instituto de Química, Universidade de São Paul, São Paulo, Brazil; 7 Genome Technology Center, Division of Advanced Research Technologies, NYU Langone Health, New York, NY USA

**Keywords:** LINE-1, Targeted PCR, Next generation sequencing

## Abstract

**Background:**

Transposable elements make up a significant portion of the human genome. Accurately locating these mobile DNAs is vital to understand their role as a source of structural variation and somatic mutation. To this end, laboratories have developed strategies to selectively amplify or otherwise enrich transposable element insertion sites in genomic DNA.

**Results:**

Here we describe a technique, Transposon Insertion Profiling by sequencing (TIPseq), to map Long INterspersed Element 1 (LINE-1, L1) retrotransposon insertions in the human genome. This method uses vectorette PCR to amplify species-specific L1 (L1PA1) insertion sites followed by paired-end Illumina sequencing. In addition to providing a step-by-step molecular biology protocol, we offer users a guide to our pipeline for data analysis, TIPseqHunter. Our recent studies in pancreatic and ovarian cancer demonstrate the ability of TIPseq to identify invariant (fixed), polymorphic (inherited variants), as well as somatically-acquired L1 insertions that distinguish cancer genomes from a patient’s constitutional make-up.

**Conclusions:**

TIPseq provides an approach for amplifying evolutionarily young, active transposable element insertion sites from genomic DNA. Our rationale and variations on this protocol may be useful to those mapping L1 and other mobile elements in complex genomes.

**Electronic supplementary material:**

The online version of this article (10.1186/s13100-019-0148-5) contains supplementary material, which is available to authorized users.

## Background

Long INterspersed Element-1 (LINE-1, L1) is one of the most abundant mobile DNAs in humans. With roughly 500,000 copies, LINE-1 sequences comprise about 17% of our DNA [1]. Although most of these exist in an invariant (fixed) state and are no longer active, about 500 insertions of the *Homo sapiens* specific L1 sequences (L1Hs) are more variable and derive from a few ‘hot’ L1Hs that remain transcriptionally and transpositionally active [2–7]. The activity of LINE-1 results in transposable element insertions that are a significant source of structural variation in our genomes [8–11]. They are responsible for new germline L1 insertion events as well as the retrotransposition of other mobile DNA sequences including *Alu* Short INterspersed Elements (SINEs) [12–15] and SVA (SINE/VNTR/*Alu*) retrotransposons [16]. Additionally, LINE-1 can propagate in somatic tissues, and somatically-acquired insertions are frequently found in human cancers [17–23].

Characterizations of transposable element sequences remain incomplete in part because their highly repetitive nature poses technical challenges. Using these high copy number repeats as probes or primer sequences can create signals or products in hybridization-based assays and PCR amplifications that do not correspond to discrete genomic loci. Moreover, both the absence of many common insertion variants from the reference genome assembly as well as the presence of hundreds of thousands of similar sequences together complicate sequencing read mappability. Detecting insertions that occur as low frequency alleles in a mixed sample presents an additional challenge, such as occurs with somatically-acquired insertions. Nevertheless, several recent studies describe strategies for mapping these elements and highlight LINE-1 continued activity in humans today. These methods include hybridization-based enrichment [24–29]; selective PCR amplification [6, 17, 30–39]; and tailored analyses of whole genome sequencing reads [10, 11, 18, 19, 40, 41].

**Fig. 1:**
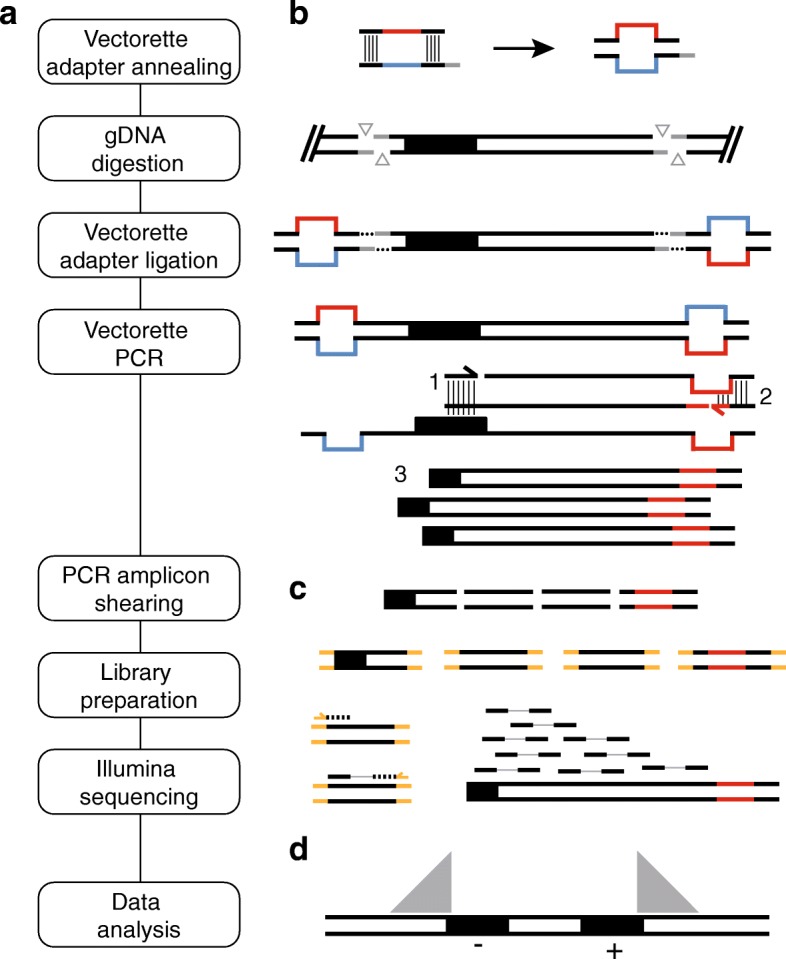
Steps in the TIPseq protocol. **a** Steps in TIPseq are shown from top to bottom in a vertical flow chart. These include (i.) vectorette adapter annealing, (ii.) genomic DNA (gDNA) digestion, (iii.) vectorette adapter ligation, (iv.) vectorette touchdown PCR, (v.) PCR amplicon shearing, (vi.) sequencing library preparation, (vii.) Illumina sequencing, and, (viii.) data analysis. The first seven of these steps are shown adjacent to schematic representations in part **b.**, to the right. **b** Vectorette adapter annealing is shown first. Mismatched sequences within the hybridized vectorette oligonucleotides are illustrated in red and blue, and create a duplex structure with imperfect base pairing. The sticky end overhang on one strand of the vectorette (here, a 5′ overhang on the bottom strand) is drawn in gray. This overhang in the annealed vectorette complements sticky ends left by genomic DNA digest, and the digest and vectorette ligations are shown in the subsequent two steps. The black box within the gDNA fragment illustrate a LINE-1 element of interest (i.e., a species-specific L1Hs). Most gDNA fragments will not have a transposable element of interest, and thus cannot be amplified efficiently by the vectorette PCR. In vectorette PCR, the L1Hs primer begins first strand synthesis (1) and extends this strand through the ligated vectorette sequence. The reverse primer complements this first-strand copy of the vectorette (2) and the two primers participate in exponential amplification (3) of these fragments in subsequent cycles. **c** Amplicons are sheared, and conventional Illumina sequencing library preparation steps complete the protocol. Paired-end sequencing reads are required to perform data analysis with TIPseqHunter. **d** A diagram of read pile-ups demonstrate how there is deep coverage of the 3′ end of L1Hs elements. For elements on the plus (+) strand with respect to the reference genome, the amplified sequences are downstream of the insertion site (i.e., covering genomic coordinates ascending from the transposon insertion). For minus (−) stranded insertions, sequences are recovered in the opposite direction

Here we present a detailed protocol to amplify and sequence human LINE-1 retrotransposon insertion loci developed in the Burns and Boeke laboratories, Transposon Insertion Profiling by sequencing (TIPseq) [22, 23, 42–44]. This method uses ligation-mediated, vectorette PCR [45] to selectively amplify regions of genomic DNA directly 3′ of L1Hs elements. This is followed by library preparation and Illumina deep sequencing (see Fig. 1a). TIPseq locates fixed, polymorphic, and somatic L1Hs insertions with base pair precision and determines orientation of the insertion (i.e., if it is on the plus (+) or minus (−) strand with respect to the reference genome). It detects, though does not distinguish between, both full length and 5′ truncated insertions as short as 150 bp. TIPseq is highly accurate in identifying somatic L1 insertions in tumor versus matched normal tissues, and allows sequencing coverage to be efficiently targeted to LINE-1 insertion sites so it is an economical way to process samples for this purpose. We have used TIPseq to demonstrate LINE-1 retrotransposition in pancreatic [22] and ovarian [23] cancers, and to show that somatically-acquired insertions are not common in glioblastomas [44]. Together with the machine learning-based computational pipeline developed in the Fenyӧ Lab for processing TIPseq data, TIPseqHunter [23], this protocol allows researchers to map LINE-1 insertion sites in human genomic DNA samples and compare insertion sites across samples.

## Results

### Experimental design

#### Starting material and optimal reaction size

High molecular weight genomic DNA is the starting material for TIPseq. This can be isolated from fresh or frozen tissues or cells. We typically use gDNA from phenol:chloroform extractions and ethanol precipitations, or from silica column preparations. This protocol uses reaction sizes producing consistent results in our hands with starting material of 10 μg genomic DNA (gDNA). We have successfully used a 3.3 μg gDNA input ‘scaled-down’ protocol with comparable results to the full-scale protocol. However, we caution that smaller reaction volumes will magnify effects of sample evaporation or slight inaccuracies in pipetting. It is important to maintain accurate reaction volumes at each step of the protocol. See Additional file 1: Table S1 for scaled-down reactions that start with as low as 3.3 μg of gDNA.

#### Restriction enzyme selection

TIPseq uses 6 different restriction enzyme digests run in parallel to maximize the portion of the genome that is cut to a PCR-amplifiable fragment in at least one of the reactions. The combination of enzymes was selected using a greedy algorithm to maximize genomic fragments 1–5 kb long. An L1Hs insertion occurring at any location in the genome is highly likely then to be represented by a fragment 1-3 kb in size in at least one of these parallel digests. This size balances informativeness and amplification efficiency; longer fragments include more sequence, but shorter fragments amplify more efficiently. For vectorette PCR to be successful, restriction enzymes should: 1) have a recognition cut site that occurs at the right genomic frequency (many 5- or 6- base pair cutters work well); 2) cut efficiently and independent of CpG methylation, 3) leave “sticky-end” overhangs for ligation of the vectorette adapters, and 4) be able to be heat inactivated. Most importantly, no restriction enzyme should cut in the retroelement insertion at any position 3′ of the forward primer sequence. This would prevent PCR amplicons from extending into unique gDNA downstream of the element.

#### Vectorette adapter design

Pairs of vectorette oligonucleotides are annealed together to form double stranded vectorette adapters (see Table 1). At one end of the vectorette, the two strands form compatible “sticky-ends” to the restriction enzyme digestion cut sites which allows for efficient adapter ligation (see Additional file 2: Table S2). The vectorette central sequence is partially mismatched such that the vectorette primer sequence is incorporated on the bottom strand, but its reverse complement is missing from the top strand. This forces first stranded synthesis to occur out of the transposable element to create the vectorette primer binding sequence. After this initial extension, exponential amplification can proceed in subsequent PCR cycles (see Fig. 1b).

**Table 1:** Vectorette oligo and primer sequences

Enzyme Vectorette Oligo Sequences (5′ to 3′)	
AseI plus	TAGAAGGAGAGGACGCTGTCTGTCGAAGGTAAGGAACGGACGAGAGAAGGGAGAG
BspHI plus	CATGGAAGGAGAGGACGCTGTCTGTCGAAGGTAAGGAACGGACGAGAGAAGGGAGAG
BstYI plus	GATCGAAGGAGAGGACGCTGTCTGTCGAAGGTAAGGAACGGACGAGAGAAGGGAGAG
HindIII plus	AGCTGAAGGAGAGGACGCTGTCTGTCGAAGGTAAGGAACGGACGAGAGAAGGGAGAG
NcoI plus	CATGGAAGGAGAGGACGCTGTCTGTCGAAGGTAAGGAACGGACGAGAGAAGGGAGAG
PstI minus	CTCTCCCTTCTCGGATCTTAACCGTTCGTACGAGAATCGCTGTCCTCTCCTTCTGCA
Common Vectorette Oligo Sequences (5′ to 3′)	
Vectorette minus	CTCTCCCTTCTCGGATCTTAACCGTTCGTACGAGAATCGCTGTCCTCTCCTTC
Vectorette plus	GAAGGAGAGGACGCTGTCTGTCGAAGGTAAGGAACGGACGAGAGAAGGGAGAG
Primer Sequences (5′ to 3′)	
L1 Primer	AGATATACCTAATGCTAGATGACACA
Vectorette Primer	CTCTCCCTTCTCGGATCTTAA

#### Specific primer selection

**Fig. 2:**
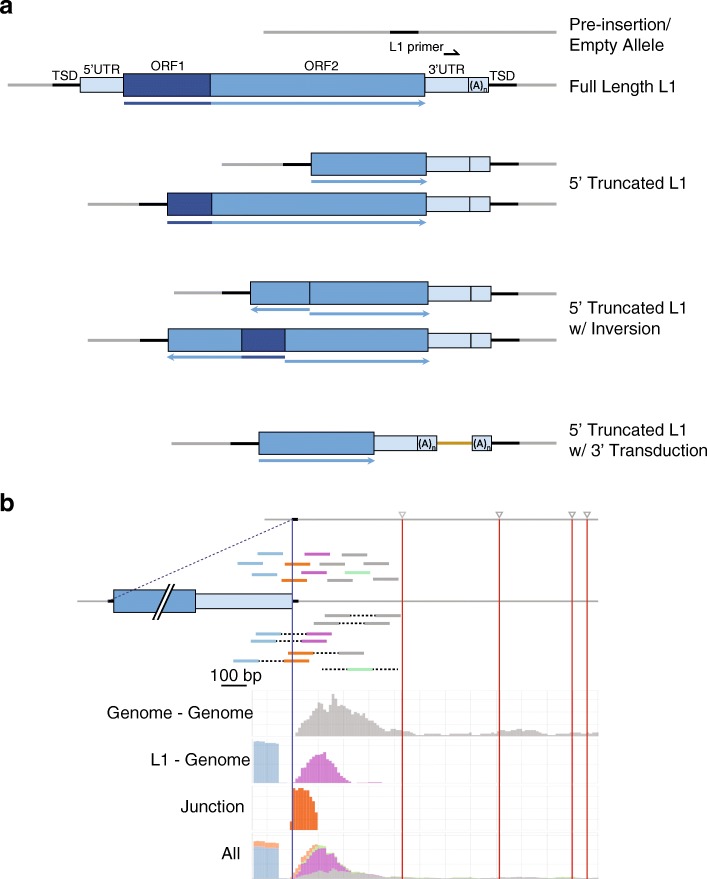
Schematic of LINE-1 and read alignments. **a** Diagrams of example LINE-1 insertions types are shown: full length, 5′ truncated, 5′ truncated with inversion, and 5′ truncated with 3′ transduction. TIPseq is able to detect these types of insertions. The full length LINE-1 element includes 5′ and 3’ UTRs, including a 3′ polyA tail, all colored in light blue. The specific L1 primer binding site is shown as a black arrow in the 3’ UTR. The open reading frames (ORF1 and ORF2) are shown in two darker shades of blue. Flanking genomic DNA is shown as gray lines with target site duplications (TSDs) as black lines. The gold line represents a transduced region of gDNA. Arrows underneath each diagram illustrate the orientation of the sequence. **b** The types of reads that TIPseq generates are shown in the top of the diagram with a TranspoScope image capture below. Reads containing only LINE-1 sequence are colored blue. Junction reads which contain both L1 and unique genomic DNA and are colored orange. Uniquely mapped genomic DNA reads are shown in gray, purple, and green. Gray reads are genome reads in genome-genome pairs. Purple reads are genome mates in genome-L1 pairs. Green reads are genome reads with an unmapped or discordant pair. TranspoScope displays the read counts and positions for specific L1 insertions detected by TIPseq. The L1 insertion site is shown as a vertical blue line, and downstream restriction enzyme cut sites used in TIPseq are shown as gray triangles with vertical red lines

The transposable element primer responsible for first strand synthesis is positioned in the 3’ UTR of the LINE-1 sequence (see Fig. 2a). The primer placement takes advantage of ‘diagnostics nucleotides’ that define currently active LINE-1. The oligo ends with the ‘ACA’ trinucleotide located in the 3’ UTR specific to the L1PA1 [also known as L1(Ta)] subset of *Homo sapiens*-specific LINE-1 (L1Hs). This strongly favors amplification of polymorphic and newly acquired somatic insertions and minimizes enrichment of older, “fixed present” elements.

#### Vectorette PCR conditions

Amplicons initiated within L1Hs insertions must traverse the LINE-1 polyA sequence and extend for a significant distance into downstream gDNA. We use a touchdown PCR program to ensure a balance between promoting primer specificity and achieving high-yields. This program progressively lowers the annealing temperature of each cycle from 72 °C to 60 °C (see Table 2). These cycling conditions, combined with the robust, proofreading DNA polymerase (ExTaq HS, Takara Bio; Shiga Japan), produces the complex mixture of optimally sized amplicons.

**Table 2:** Vectorette PCR thermal cycler program

95 °C	5 min	
95 °C	1 min	5 cycles
72 °C	1 min	
72 °C	5 min	
95 °C	1 min	5 cycles
68 °C	1 min	
72 °C	5 min	
95 °C	45 s	15 cycles
64 °C	1 min	
72 °C	5 min	
95 °C	45 s	15 cycles
60 °C	1 min	
72 °C	5 min	
72 °C	15 min	
16 °C	Hold	

#### DNA shearing

We use a Covaris focused ultrasonicator (Covaris; Woburn, MA) with the manufacturer’s recommended settings to shear the vectorette PCR amplicons to 300 bp prior to library preparation (see Additional file 3: Figure S2B). Shearing PCR amplicons may produce a broader size range than when shearing genomic DNA. If necessary, the treatment time may be modified on a per sample basis to adjust the final size distribution.

#### Library preparation and size-selection

Library construction may be performed using any kit that is compatible with Illumina next generation sequencing, including Illumina’s TruSeq LT or PCR-free DNA sample prep kits (Illumina; San Diego, CA). We recommend using Kapa Library Preparation Kit for Illumina (Kapa Biosystems; Wilmington, MA) and to follow the manufacturer’s instructions. If necessary, amplification may be performed during library construction, however, we advise using a PCR-free library preparation. Library adapters add approximately 120 bp of length to the sheared DNA. It may be necessary to perform a size selection during library preparation so that final library size is greater than 400 bp. This will prevent the generation of overlapping read pairs and reads containing adapter sequence. If necessary, we recommend performing dual-SPRI bead selection during library preparation or adding Pippin prep selection (Sage Science; Beverly, MA) after library pooling to remove all fragments smaller than 400 bp.

#### Illumina sequencing

Our data analysis pipeline, TIPseqHunter, requires 150 bp or shorter paired-end reads for optimal results. Longer reads may be trimmed to meet this requirement. We recommend a minimum of 15–25 million read pairs per sample. For example, for the Illumina HiSeq4000 this corresponds to pooling 12 samples per lane in high-output mode. These guidelines should result in sufficient coverage and read depth for identifying L1 insertion loci.

#### Data analysis

TIPseq produces reads that contain LINE-1 sequence, adjacent genomic sequence, or both (junction reads) (see Fig. 2b). TIPseq data analysis reveals precise, base-pair resolution of L1Hs insertions and their orientation). We recommend using our custom bioinformatics program: TIPseqHunter [23]. We developed this program with a machine learning algorithm that uses known insertions as a training set for identifying new insertions. TIPseqHunter is available for download at: https://github.com/fenyolab/TIPseqHunter (see Table 6). It is also available as a Docker image at: https://github.com/galantelab/tipseq_hunter. This encapsulates all java dependencies, read aligners, genome indexes and biological annotation files needed by both steps of the pipeline. The genome indexes and annotation files in both TIPseqHunter and the Docker image use the human reference genome assembly GRCh37 (hg19). Instructions for use and download can be found in the README file at: https://github.com/galantelab/tipseq_hunter/blob/master/README.md. For sequencing runs of less than 20 million read pairs, 10–20 GB of RAM is suggested, and running time using 8 core processors on a Linux system is approximately 25 h. For runs in excess of 60 million reads, TIPseqHunter requires 40–50 GB of RAM, and running time is 1–1.5 h per 1 million reads. TranspoScope, a bioinformatics tool for browsing the evidence for transposable element insertions into the genome by visualizing sequencing read coverage in regions flanking de novo insertion of transposable elements which are not present in the reference genome. TranspoScope can be downloaded at https://github.com/FenyoLab/transposcope and an instructional video is available at: https://www.youtube.com/watch?v=exVAnoMRLSM .

## Discussion

### De novo insertion validation

**Fig. 3:**
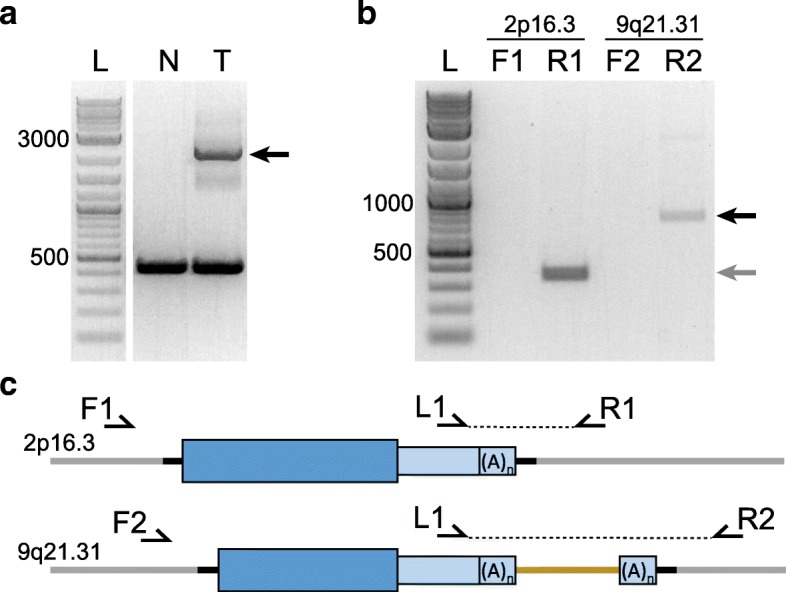
Approaches to PCR validation of insertions. **a** Agarose gel electrophoresis of a somatic PCR validation. Three lanes are shown: [L] 2-log ladder (NEB), [N] normal DNA, [T] tumor DNA. An upper band marked by a black arrow is present in the tumor but absent in the normal sample which confirms a somatic L1 insertion occurred in the tumor. **b** Agarose gel of two L1 3’ PCR validations. Five lanes are shown: [L] 2-log ladder (NEB), [F1] forward primer with L1 primer for insertion on 2p16.3, [R1] reverse primer with L1 primer for insertion on 2p16.3, [F2] forward primer with L1 primer for insertion on 9q21.31, [R2] reverse primer with L1 primer for insertion on 9q21.31. For both insertions, only the reverse primer produces a band when paired with the L1 primer, which suggests that both are plus strand insertions. All specific primers were designed approximately 200 bp away from the insertion site. Because the L1 primer is located 150 bp away from the 3′ end of the element, the expected product size for both reactions is approximately 350 bp marked with a gray arrow. The PCR reaction for the 9q21.31 insertion produces a band larger than expected marked with a black arrow. This suggests that a 3′ transduction may have taken place and is confirmed by sending the PCR product for Sanger sequencing. **c** The illustration shows the relative positions of primers and products for the two L1 insertions from part b. The 9q21.31 insertion in the lower diagram has a 3′ transduction shown as a gold line

TIPseqHunter accurately detects fixed, polymorphic, and de novo L1Hs insertions. Our previous studies have produced validation rates has high as 96% [23]. While users can therefore be confident in TIPseqHunter calls, we recommend validating at least subsets of predicted insertions whenever important conclusions are being drawn from a study. This can be accomplished by site-specific, spanning PCR and Sanger sequencing (see Table 7). This will confirm the presence of the insertion and report the length and structure of the element. It is important to use the same high quality gDNA used in the TIPseq procedure to validation insertion candidates. Normal control DNA should be tested in parallel when validating somatic insertions from tumor-normal studies (see Fig. 3a). L1-specific 3’ PCR may be used to validate large insertions that are difficult to span in PCR and to identify possible 3′ transduction events (see Table 8).

### Level of expertise required

The first part of the TIPseq protocol and final validations (steps 1–21,31) require basic molecular biology equipment and techniques (digestion, ligation, and PCR). The second part of the protocol (steps 22–29) involves the use of more advanced equipment and methods (DNA shearing, library preparation, and deep sequencing). It is possible to contract ‘advanced’ steps to sequencing core facilities depending on each user’s level of expertise and access to the required equipment, and this is our recommendation for users without training or experience with library preparation and deep sequencing. Data analysis (step 30) using TIPseqHunter and visualization using TranspoScope requires basic knowledge of NGS related bioinformatics and UNIX shell scripting experience in order to run the program from command line.

### Applications of the method

TIPseq was initially adapted from a microarray based approach called Transposon insertion profiling by microarray or TIPchip [9, 42], which was first developed for mapping Ty1 elements in *Saccharomyces cerevisae* [42] . Although TIPseq is applicable to other transposable elements or species, this protocol is optimized to detect LINE-1 insertions in the human genome, and currently our TIPseqHunter program can only process human LINE-1 TIPseq data. TIPseq may be used for a variety of applications, including: population studies to identify common structural variants, tumor vs. normal comparisons to identify somatically-acquired insertions and trace cellular phylogenies, and in patients with specific phenotypes to evaluate for de novo retrotransposition events. Whole genome sequencing (WGS) can also be used for these purposes, and the main advantage of TIPseq is that insertion sites can be relatively deeply sequenced inexpensively. Targeting sequencing to retrotransposon insertion sites can result in a 400x cost saving for L1Hs mapping, and a 60x cost savings for *Alu* mapping.

### Limitations of the method

Although TIPseq is a highly useful tool for detecting LINE-1 insertions, there are some limitations to the method that should be considered. First, TIPseq relies on restriction enzyme digestion of a large amount of high quality (high molecular weight) genomic DNA. For samples with limited amounts or reduced quality DNA, such as single-cell or fixed tissue, this protocol may need adjusted to work with similar efficiency. Secondly, while this method provides insertion location and orientation information, it does not differentiate between insertion ‘types’. This includes classifying full length versus truncated insertions and elements with 5′ inversions or 3′ transductions (see Fig. 2a). While TIPseq will detect these insertions, further analysis, such as gel electrophoresis or Sanger sequencing, is required to confirm insert size and sequence variations. Finally, TIPseq does not distinguish between heterozygous and homozygous insertion alleles. An additional qualitative validation, such as PCR, is needed to confirm zygosity.

### Anticipated results

The TIPseq procedure should yield more than 10 μg of purified PCR amplicons depending on vectorette PCR efficiency. The size distribution of these amplicons usually averages 1-3 kb (see Additional file 4: Figure S1A). This size distribution may vary depending on the quality of starting material. Sheared DNA should average around 300 bp (see Additional file 3: Figure S2B). Shearing of PCR amplicons produces a broader size range than when shearing gDNA. If necessary, shearing conditions may be adjusted to alter the final size distribution. The HiSeq4000 generates approximately 300 million read pairs per lane. Pooling up to 12 samples per lane will produce the recommended minimum of 15–25 million read pairs per sample. The final sequencing output consists of reads that align to the 3’UTR of LINE-1 and/or the adjacent genomic DNA. Read pairs will be either L1-genome, genome-genome, L1-junction, or junction-genome, or ‘unpaired’ genome (see Fig. 2b). On average, approximately 30 to 40% of TIPseq reads will align to LINE-1 sequence. Our validation rates for detecting new L1 insertions are as high as 96% [23]. TIPseq will identify full length and 5′ truncated L1’s 150 bp and larger, including elements with 5′ inversions and 3′ transductions. However, additional PCR and Sanger sequencing must be performed to confirm these events (see Table 8).

## Conclusions

This protocol describes in detail our approach to transposon insertion profiling by next-generation sequencing (TIPseq). The assay as described targets signature sequences in the 3’UTR of evolutionarily young L1PA1 elements for insertion site amplification. A subset of these elements is active in the modern human genome. Their ongoing activity makes them valuable to map for characterizing heritable genetic polymorphisms, de novo insertions, and somatic retrotransposition activity. While LINE-1 insertion sites can be detected in whole genome sequencing data, selectively amplifying these sites can allow investigators to target their sequencing to insertion locations. This enables LINE-1-directed studies to more efficiently and affordably use sequencing and computational resources. We have demonstrated that variations of this protocol are effective at selectively amplifying other transposable element in humans [i.e., *Alu* insertions (See Additional file 5: Table S3), and endogenous retroviruses (ERV-K)], and we expect that similar approaches can be taken to map active mobile genetic elements, other high-copy recurrent sequences, or transgene insertions.

## Methods

### Reagents

- Molecular biology grade water (Corning, cat. no. 46–000-CM)
- Oligonucleotides and primers (IDT), see Table 1
- 25 mM MgCl2 (Life Technologies, cat. no. R0971)
- 10 mM Tris-EDTA (TE) buffer, pH 8.0 (Quality Biological, cat. no. 351–011-131)
- 1 M Tris-HCl buffer, pH 8.0 (Quality Biological, cat. no. 351–007-101)
- Ethanol, Absolute (200 Proof), Molecular Biology Grade (Fisher Scientific, cat. no. BP2818500) (CAUTION Ethanol is highly flammable)
- *Ase*I (NEB, cat. no. R0526S)
- *BspH*I (NEB, cat. no. R0517S)
- *BstY*I (NEB, cat. no. R0523S)
- *Hind*III (NEB, cat. no. R0104S)
- *Nco*I (NEB, cat. no. R0193S)
- *Pst*I (NEB, cat. no. R0140S)
- RNase cocktail enzyme mix (Life Technologies, cat. no. AM2286)
- T4 DNA ligase (NEB, cat. no. M0202S)
- Adenosine 5′-Triphosphate, ATP (NEB, cat. no. P0756S)
- TaKaRa Ex Taq DNA polymerase, Hot-Start (Clontech, cat. no. RR006A)
- QiaQuick PCR Purification Kit (Qiagen, cat. no. 28106)
- Zymoclean Gel DNA Recovery Kit (Zymo Research, cat. no D4002)
- Ultrapure Agarose (Life Technologies, cat. no. 16500–100)
- Gel Loading Dye, 6x (NEB, cat. no. B7022S)
- UltraPure Tris-Acetate-EDTA (TAE) buffer, 10x (Life Technologies, cat. no. 15558–026)
- Ethidium Bromide solution, 10 mg/mL (Bio-Rad, cat. no. 161–0433) (CAUTION Ethidium bromide is toxic and is a potential mutagen and carcinogen.)
- 2-log ladder (NEB, cat. no. N3200S)
- Qubit dsDNA HS assay kit (ThermoFisher Scientific, cat. no. Q32851)
- Agilent DNA 1000 kit (Agilent, cat. no. 5067–1504)
- Agencourt AMPure XP Magnetic Beads (Beckman Coulter, cat. no. A63882)
- KAPA HTP Library Preparation Kit for Illumina (KAPA Biosystems, cat. no. KK8234).
- KAPA Library Quantification Kit, complete kit, universal (Kapa Biosystems, cat. no. KK4824)
- PhiX Control v3 (Illumina, cat. no. FC-110-3001)
- HiSeq 3000/4000 SBS Kit, 300 cycles (Illumina, cat. no. FC-410-1003)
- Pippin Prep DNA gel cassettes, 2% agarose (Sage Science, cat. no. CEF2010)

### Equipment

- 1.7 mL microcentrifuge tubes (Denville, cat. no. C2170)
- 0.2 mL PCR 8-Strip tubes (Midsci, cat. no. AVSST)
- Eppendorf Microcentrifuge 5424 (Eppendorf, cat. no. 5424 000.614)
- Eppendorf fixed-angle rotor (Eppendorf, cat. no. 5424 702.007)
- Digital Incublock (Denville, cat. no. I0520)
- Modular block (Denville, cat. no. I9013)
- Applied Biosystems Thermal Cycler 2720 (Life Technologies, cat. no. 4359659)
- NanoDrop™ 8000 Spectrophotometer (ThermoFisher Scientific, cat. no. ND-8000-GL)
- Electrophoresis gel system (USA Scientific, cat. no. 3431–4000)
- Electrophoresis power supply (Fisher Scientific, cat. no. S65533Q)
- Qubit Fluorometer (ThermoFisher Scientific, cat. no. Q33226)
- Qubit assay tubes (ThermoFisher Scientific, cat. no. Q32856)
- Agilent 4200 TapeStation (Agilent, cat. no. G2991AA)
- High sensitivity D1000 ScreenTape (Agilent, cat. no. 5067–5584).
- High sensitivity D1000 Reagents (Agilent, cat. no. 5067–5585).
- Covaris LE220 Focused-ultrasonicator and chiller (Covaris, model no. LE220)
- Covaris microTUBEs (Covaris, cat. no. 520052)
- Covaris microTUBE rack (Covaris, cat. no. 500282)
- DynaMag-2 magnetic rack (Life Technologies, cat. no. 12321D)
- HiSeq 4000 System (Illumina)
- Pippin Prep DNA Size Selection System (Sage Science, cat. no. PIP0001)
- CFX96 Touch Real-Time PCR Detection System (BioRad, cat. no. 1855195)

### Reagent setup

#### Genomic DNA

TIPseq requires starting with high molecular weight genomic DNA. We recommend isolating fresh gDNA when possible. Poor quality genomic DNA will reduce TIPseq’s efficiency. Always avoid vortexing, rough pipetting, and excessive freeze-thaw cycles to ensure gDNA integrity is maintained throughout the protocol.

#### Oligonucleotide stocks

Vectorette adapter oligonucleotides should be resuspended with TE buffer to stock concentrations of 100 μM. PCR primers should be resuspended with molecular grade water to stock concentrations of 100 μM. Stocks should be stored at − 20 °C, thawed and mixed well before use.

#### Master mix preparations

All master mixes should be prepared on ice immediately before use. We recommend including a 2–3 sample excess when preparing each master mix. See Tables 3, 4, 5 for master mix formulas.

**Table 3:** Digestion master mix

Digestion master mix	Volume (μL)	
	1x	4x
Molecular grade H_2_O	2.25	9.0
10x Restriction enzyme buffer	2.5	10
Restriction enzyme	1.0	4.0
RNase cocktail enzyme mix	0.25	1.0

**Table 4:** Ligation master mix

Ligation master mix	Volume (μL)	
	1x	8x
10 mM ATP	2.5	20
10x T4 Ligase buffer	0.5	4.0
T4 Ligase (400 U/μL)	0.2	1.6

**Table 5:** PCR master mix formulas

PCR master mix	Volume (μL)	
	1x	8x
Molecular grade H_2_O	32.55	260.4
10x Ex Taq buffer	5.0	40
dNTP mixture (2.5 mM each)	4.0	32
Specific L1 Primer (100 μM)	0.1	0.8
Vectorette Primer (100 μM)	0.1	0.8
Ex Taq HS polymerase	0.25	2.0

### Equipment setup

#### Thermal cycler

We recommend performing the restriction enzyme digestions, inactivation steps, and PCR in a pre-heated thermal cycler with heated lid.

#### Agarose gel electrophoresis

DNA and ladder is loaded into a 1% agarose/1x TAE gel pre-stained with ethidium bromide (1:20,000 dilution). (CAUTION Ethidium bromide is toxic and is a potential mutagen and carcinogen. Use proper protective wear.) The gel should be run at a constant 100 V for 45 min or until separation of the ladder is clearly visible.

#### Covaris shearing system

The Covaris LE220 shearing system is setup according to the manufacturer’s instructions.

## Procedure

### Steps 1–5: Vectorette adapter annealing (Timing: 2 h)

1. In a 1.7 mL tube add 20 μL of 100 μM vectorette oligo stock to 300 μL TE buffer to make 6.25 μM working concentrations of all vectorette oligos.
2. Add 32 μL of a 6.25 μM enzyme vectorette oligo and 32 μL of a 6.25 μM common vectorette oligo to 28 μL of TE buffer. Incubate at 65 °C in heat block for 5 min.
  **Critical:** Always combine a plus and a minus oligo together and always combine an enzyme vectorette oligo with a common vectorette oligo (See Table 1)
3. Add 8 μL of 25 mM MgCl_2_. Pipet well to mix. Incubate at 65 °C in heat block for 5 min.
4. Keeping tubes in block, remove block from heat, and allow to slowly come to room temperature.
5. Add 100 μL of TE buffer to bring the final concentration of the vectorette adapters to 1 μM.
  **Pause Point**: Annealed vectorette adapters should be stored at − 20 °C.

### Steps 6–9: Genomic DNA digestion (Timing: 1 h setup and overnight incubation)

6. Dilute 10 μg genomic DNA in 123.5 μL of molecular grade water and aliquot diluted gDNA to each of six 0.2 mL PCR tubes
7. Prepare digestion master mix on ice for the appropriate number of samples plus excess (See Table 3). Mix by gently pipetting the entire volume 5 times and quickly spin to collect.
8. Add 6 μL of digestion master mixes in parallel to each gDNA aliquot. Mix by gently flicking and spinning.
9. Incubate overnight at the appropriate activation temperature in a thermal cycler with heated lid.

### Steps 10–14: Vectorette adapter ligation (Timing: 3 h setup and overnight incubation)

10. Inactivate the restriction enzyme digests for 20 min at 80 °C in thermal cycler with heated lid. Cool to room temperature.
11. Add 2 μL of the appropriate 1 μM annealed vectorettes adapters to each digest and mix by gently flicking and spinning.
  **Critical:** Be sure to add each annealed vectorette to its corresponding enzyme digest.
12. Use a thermal cycler with heated lid to incubate at 65 °C for 5 min and then slowly cool to room temperature (0.5 °C/min). Move samples to 4 °C for at least 1 h.
13. Prepare ligation master mix on ice for the appropriate number of samples plus excess (See Table 4). Mix by gently pipetting the entire volume 5 times and quickly spin to collect.
14. Add 3.2 μL of ligation master mix to the 6 enzyme/vectorette tubes. Mix by gently flicking and spinning. Keep at 4 °C overnight.

### Steps 15–18: Vectorette PCR (Timing: 1 h setup and 7 h runtime)

15. Inactivate ligation reactions by incubating at 65 °C for 20 min in a thermal cycler with heated lid.
  **Pause Point:** The vectorette-ligated DNA templates may be kept at 4 °C for short term or − 20 °C for long term storage.
16. Prepare PCR master mix on ice for the appropriate number of samples plus excess (See Table 5). Mix by gently pipetting the entire volume 5 times and quickly spin to collect.
17. Add 42 μL of PCR master mix to 8 μL of each vectorette-DNA template (and to 8 μL of H_2_O for a no-template control). Mix by gently flicking and spinning.
  **Critical:** Be sure to set up 6 separate PCR reactions for each of the 6 DNA-vectorette templates. Only part of the DNA template may be used, and the remainder can be kept at 4 °C for short term or − 20 °C for long term storage.
18. Run vectorette PCR program in thermal cycler with heated lid (see Table 2). The program can be left to run overnight.

### Steps 19–21: DNA purification and quality control (Timing: 2 h)

19. Purify PCR reactions using 1x volume of Agencourt AMPure beads. Elute in 20uL 10 mM Tris-HCL pH 8.0 and pool together.
  **Pause Point:** Purified DNA may be kept at 4 °C for short term or − 20 °C for long term storage.
20. Measure purified DNA concentration on NanoDrop.
  **Troubleshooting**: If PCR yield is too low, restart procedure with freshly annealed vectorette adapters, isolate fresh gDNA, or increase the initial amount of gDNA.
21. Run 2 μg of purified DNA on 1.5% agarose gel.
  **Critical:** Vectorette PCR amplicons should appear as a smear on the gel averaging around 1-3 kb. (see Additional file 4: Figure S1A).
  **Troubleshooting:** The presence of a very high molecular weight smear could indicate primer-vectorette concatemer amplification. Digest 2 μg of purified vectorette PCR amplicons with *Bst*YI and run on a 1.5% agarose gel. *Bst*YI cuts within the vectorette primer. An intense band around 50 bp indicates the presence of vectorette-primer concatemers in the PCR product (see Additional file 4: Figure S1B).

### Steps 22–25: DNA shearing and purification (Timing: 2 h)

22. Based on NanoDrop measurement, prepare 10 μL of 100 ng/μL purified DNA in H_2_O. Measure diluted DNA concentration on Qubit.
23. Based on the Qubit measurement, dilute 1.5 μg of purified DNA in 130 μL 10 mM Tris-HCL and transfer to a Covaris microTUBE.
  **Critical**: The Qubit is more reliable than the NanoDrop at measuring double-stranded DNA concentration.
24. Shear DNA to 300 bp using Covaris’ LE220 with recommended settings: duty factor = 30%, peak incident power = 450, cycles/burst = 200, time = 60s
25. Purify sheared DNA using QiaQuick PCR Purification kit. Elute in 50 μL H_2_O.
  **Pause Point:** Sheared DNA may be kept at 4 °C for short term or − 20 °C for long term storage.
  **QC (Optional):** Run sheared DNA on Agilent 4200 TapeStation. The trace should show a peak centered around 300 bp (see Additional file 3: Figure S2B).

### Steps 26–28: Library preparation and quality control (Timing: 1 d)

26. Use 200 ng of sheared DNA to prepare libraries using KAPA Library Preparation Kit for Illumina according to the manufacturer’s instructions without performing dual-SPRI size selection.
  **Critical:** Avoid performing library amplification. We recommend avoiding size selection, but dual-SPRI bead selection may be performed.
  **Pause Point:** Libraries may be stored at − 20 °C.
27. Perform QC on prepared libraries using qubit and Agilent 4200 TapeStation.
  **Troubleshooting**: If library yield is too low, restart library preparation with more sheared DNA (0.5–1 μg). If necessary, perform qPCR on prepared libraries with KAPA Library Quantification Kit to increase accuracy of quantification and pooling.
28. If necessary, appropriately pool samples to create a multiplexed library.
  **Critical**: Pool up to 12 samples per lane to get a minimum of 15–25 million read pairs per sample.
  **Troubleshooting:** Performing qPCR on prepared libraries with KAPA Library Quantification Kit prior to pooling may result in a more balanced sequencing output.

### Steps 29: Illumina deep sequencing (Timing: 1–4 d)

29. Sequence 200pM of pooled library with 20% PhiX on Illumina HiSeq4000, 150 cycles, paired end. If necessary, demultiplex raw reads.

### Steps 30–31: Data analysis and validation (Timing: Variable)

30. Analyze data using TIPseqHunter (see Table 6).
  **Troubleshooting:** If the data contain a large amount of overlapping read pairs, use Pippin prep selection after pooling (step 28) to remove fragments under 400 bp.
31. Perform PCR validation and Sanger sequencing (see Tables 7 and 8)

**Table 6:** Data analysis using TIPseqHunter (Timing: variable)

TIPseqHunter uses genome assembly GRCh37 (hg19) and can be run with a Docker image or by using individual programs. TIPseqHunter was developed by Java (version 7) and R (version 3.2) languages and tested under Linux operating system and is available to download at: https://github.com/fenyolab/TIPseqHunter Docker image for TIPseqHunter was developed with the stable version of Docker Community Edition (CE) and it may work under any operating system capable to run Docker. However, we recommend the Unix-like operating systems, such as Linux and Mac OS X. Our Docker image is an alternative to the conventional TIPseqHunter program mentioned above. This image version is available at Docker Hub registry (https://hub.docker.com/) and can be downloaded with the Docker client command: *docker pull galantelab/tipseqhunter*. For further details, check https://github.com/galantelab/tipseq_hunter/blob/master/README.md Testing data and masked and bowtie-built reference genome are available to download at: http://openslice.fenyolab.org/data/tipseqhunter/test_data **Docker Prerequisite:** The Docker image works as a container and runs exactly the same TIPseqHunter program. Neither downloading of dependencies nor manually setting of software used by TIPseqHunter are required. In order to run this container you will need only need to install Docker. For OS X: https://docs.docker.com/mac/started/ For Linux: https://docs.docker.com/linux/started/ For Windows: https://docs.docker.com/docker-for-windows/ **TIPseqHunter Prerequisites:** 1. At least 10GB of memory is needed if the number of sequenced read-pairs is greater than 20M. 2. Bowtie 2 alignment software (version 2.2.3 used for testing): http://bowtie-bio.sourceforge.net/bowtie2/index.shtml 3. Samtools software (latest version): http://samtools.sourceforge.net/ 4. Trimmomatic software (version 0.32 used for testing): http://www.usadellab.org/cms/?page=trimmomatic 5. Java packages: sam-1.112.jar, commons-math3-3.4.1.jar, jfreechart-1.0.14.jar, jcommon-1.0.17.jar, itextpdf-5.2.1.jar, biojava3-core-3.0.1.jar 6. R packages: pROC, ggplot2, caret, e1071 **Critical:** BAM file has to be generated by bowtie2 alignment with "XM" tag **Running TIPseqHunter:** **(1) for quality control, alignment, feature selection, modeling, prediction:** ./TIPseqHunterPipelineJar.sh fastq_path output_path fastq_r1 key_r1 key_r2 num_rp **Critical**: Detailed information is provided in the TIPseqHunterPipelineJar.sh file. Some parameters need to be pre-set. Parameters: fastq_path: path of the fastq files (Note: this is the only path and file name is not included) output_folder: path of the output files (Note: this is the only path and file name is not included) fastq_r1: read 1 file name of paired fastq files key_r1: key word to recognize read-1 fastq file (such as "_1" is the key word for CAGATC_1.fastq fastq file) **Critical:** key has to be unique in the file name key_r2: key word to recognize read-2 fastq file and replaceable with the read-1 key word to match to read-1 file (such as "_2" is the key word for CAGATC_2.fastq fastq file) **Critical:** key has to be unique in the file name num_rp: the total number of the read pairs in the paired fastq files (Note: it is the total number of read-pairs, i.e. either the total number of read1 or read2 but not together.) (This number is for normalization purpose) **(2) for somatic insertions:** TIPseqHunterPipelineJarSomatic.sh repred_path control_path repred_file control_file **Critical**: Detailed information is provided in the TIPseqHunterPipelineJarSomatic.sh file. Some parameters need to be pre-set. Parameters: repred_path: path of “model” folder under output folder control_path: path "TRLocator" folder under output folder repred_file: file with suffix ".repred" and generated from P11 in repred_path (Note: file name should be ending with ".repred".) (such as 302_T_GTCCGC.wsize100.regwsize1.minreads1.clip1.clipflk5.mindis150.FP.uniqgs.bed.csinfo.lm.l1hs.pred.txt.repred) control_file: file with suffix “.bed” in control_path (Note: file name should be ending with ".bed".) (such as 302_N_GTGAAA.fastq.cleaned.fastq.pcsort.bam.w100.minreg1.mintag1

**Table 7:** Validation of insertions through spanning PCR and Sanger sequencing (Timing: variable)

1. Design flanking primers around L1 insertion site. **Critical**: Each primer should be at least 100bp away from insertion site. Avoid placing primers in repetitive DNA. 2. Set up 25uL PCR reactions with ExTaq HS following manufacturer’s instructions. **Critical**: It is important to use a robust polymerase to extend through the L1 poly-A tail. 3. Use 50ng of gDNA as template. **Critical**: Use the same high quality gDNA that served as starting material for TIPseq 4. Run the PCR in a thermal cycler with heated lid using a 10-minute extension for 30 cycles. **Critical:** It is necessary to use an extension time long enough to amplify a full length, 6kb L1 insertion. 5. Run the PCR product on a 1% agarose gel and excise the band containing the filled allele (see Fig. 3a). **Troubleshooting**: If no filled band occurs, we recommend trying a 3’ L1 specific PCR. (see Table 8). 6. Purify the excised DNA using Zymoclean Gel DNA Recovery Kit following the manufacturer’s instructions. 7. Sanger sequence the purified DNA using both the forward and reverse PCR primer

**Table 8:** Validation of insertions and identification of 3’ transduction events through L1-specific 3’ PCR and Sanger sequencing (Timing: variable)

1. Design flanking primers around L1 insertion site. **Critical:** Each primer should be at least 100bp away from insertion site. Avoid placing primers in repetitive DNA. 2. Set up duplicate 25uL PCR reactions with ExTaq HS following manufacturer’s instructions. Each reaction should contain one flanking primer paired with the L1 specific primer from vectorette PCR. **Critical:** It is important to use a robust polymerase to extend through the L1 poly-A tail. 3. Use 50ng of gDNA as template. **Critical:** Use the same high quality gDNA that served as starting material for TIPseq 4. Run the PCR in a thermal cycler with heated lid using a 60°C annealing temperature and at least a 30-second extension for 30 cycles. **Critical:** It is important to use a slightly higher annealing temperature and shorter extension time to reduce the amount of off-target L1 binding and amplification. 5. Run the PCR product on a 1% agarose gel and excise the band from the successful reaction. **Critical**: Only one of the two PCR reactions should produce a band. A plus stranded L1 insertion will produce a band in the reverse primer reaction, and a minus stranded L1 will produce a band in the forward primer reaction. The size of the band should equal the distance from the genomic primer to the L1 insertion site plus 150bp of L1 and polyA sequence. A band larger than expected could indicate a 3’ transduction event has occurred (See Fig. 3b) 6. Purify the excised DNA using Zymoclean Gel DNA Recovery Kit following the manufacturer’s instructions. 7. Sanger sequence the purified DNA using the L1 primer and either the forward or the reverse genomic primer, depending on which reaction was successful. **Critical:** It may be necessary to use internal primers to sequence through the product completely.	

## Timing

- Steps 1–5, vectorette adapter annealing: 2 h
- Steps 6–9, genomic DNA digestion: 1 h setup and overnight incubation
- Steps 10–14, vectorette adapter ligation: 3 h setup and overnight incubation
- Steps 15–18, vectorette PCR: 1 h setup and 7 h runtime
- Steps 19–21, DNA purification and quality control: 2 h
- Note: Waiting and processing time varies when sending PCR amplicons to a sequencing core facility.
- Steps 22–25, DNA shearing and purification: 1 h
- Steps 26–28, library preparation and quality control: 1 d
- Step 29, Illumina deep sequencing: 1–4 days
- Steps 30–31, Data analysis and validation: variable
- Table 6, Data analysis using TIPseqHunter: variable
- Table 7, Validation of insertions through spanning PCR and Sanger sequencing: variable
- Table 8, Validation of insertions and identification of 3′ transduction events through L1-specific 3’ PCR and Sanger sequencing: variable
- Note: Sequencing, analysis, and validation time will vary depending on the number of samples being processed and number of insertions to validate.

## Troubleshooting

See Table 9 for troubleshooting information.

**Table 9:** Troubleshooting table

Step	Problem	Possible reason	Solution
20	Low PCR yield	Poor vectorette adapter annealing or ligation	Anneal fresh vectorette adapters and repeat procedure
20	Low PCR yield	Low starting gDNA quality/quantity	Increase the initial amount of starting gDNA, or isolate fresh gDNA
21	Very high molecular weight smear	Vectorette-Primer concatemer amplification	Digest 2 μg of vectorette PCR amplicons with BstYI and running on a 1.5% agarose gel. An intense band around 50 bp indicates the presence of concatemers in the PCR product (see Additional file 4: Figure S1B). Repeat procedure with fresh reagents in an amplification-free area.
27	Library yield too low to sequence	DNA lost during library preparation or size-selection	Restart library preparation with more sheared DNA (0.5-1 μg)
28	Uneven sequencing output distribution	Uneven library pooling	Performing qPCR on prepared libraries with KAPA Library Quantification Kit prior to pooling may result in a more balanced sequencing output.
30	High number of overlapping read pairs	Small library fragments	Add a Pippin prep selection after pooling (step 28) to remove fragments under 400 bp.
Table 7, step 5	No L1 insertion band	Large/difficult L1 insertion	Use L1-specific 3’ PCR (see Table 8)

## Additional files

Additional file 1: **Table S1.** Scaled-down reaction sizes. (XLSX 13 kb)

Additional file 2: **Table S2.** Design of the vectorette oligo and primer sequences. (XLSX 12 kb)

Additional file 3: **Figure S2.** DNA size distributions during TIPseq. **a.** An Agilent TapeStation image of two samples of purified vectorette PCR DNA is shown with amplicons averaging 1-3 kb. The protocol does not require running samples on TapeStation after vectorette PCR, but this image is included to illustrate the size range. **b.** The second TapeStation image shows the samples after DNA shearing. The average size distribution for the sheared DNA should be approximately 300 bp. **c.** As a final quality control, samples should be run on the TapeStation after library prep is completed. This image shows the increase in size from the Illumina adapters and an average library size around 400 bp. (PDF 952 kb)

Additional file 4: **Figure S1.** Vectorette PCR amplicons quality control. **a.** Electrophoresis of vectorette PCR amplicons. The gel image shows five lanes: [L] 2-log ladder (NEB) [1], 2 μg purified PCR amplicons [2], 2 μg purified PCR amplicons digested with *Bst*YI [3], 2 μg purified PCR amplicons ‘contaminated’ with concatemers [4], 2 μg purified PCR amplicons digested with *Bst*YI showing concatemer band at ~ 50 bp. A good vectorette PCR will present as a smear of amplicons averaging 1-3 kb (lane 1). A very high molecular weight smear could indicate contamination (lane 3). **b.** Schematic of possible vectorette-primer concatemer. Digestion of the amplicons with *Bst*YI, which cuts inside the vectorette primer sequence, will produce a visible band at ~ 50 bp if concatemers are in the PCR product. (PDF 2479 kb)

Additional file 5: **Table S3.** Retrotransposon primer sequences. (XLSX 8 kb)

## Abbreviations

L1Hs: *Homo sapiens*-specific L1
LINE-1, L1: Long interspersed element-1
TIP: Transposon insertion profiling
