# Supplementary figures and images for: Transposon insertion profiling by sequencing (TIPseq) for mapping LINE-1 insertions in the human genome
Source: Mob DNA. 2019 Mar 8;10:8. doi: 10.1186/s13100-019-0148-5 (PMC6407172; doi:10.1186/s13100-019-0148-5)

Figure S2 (Burns)

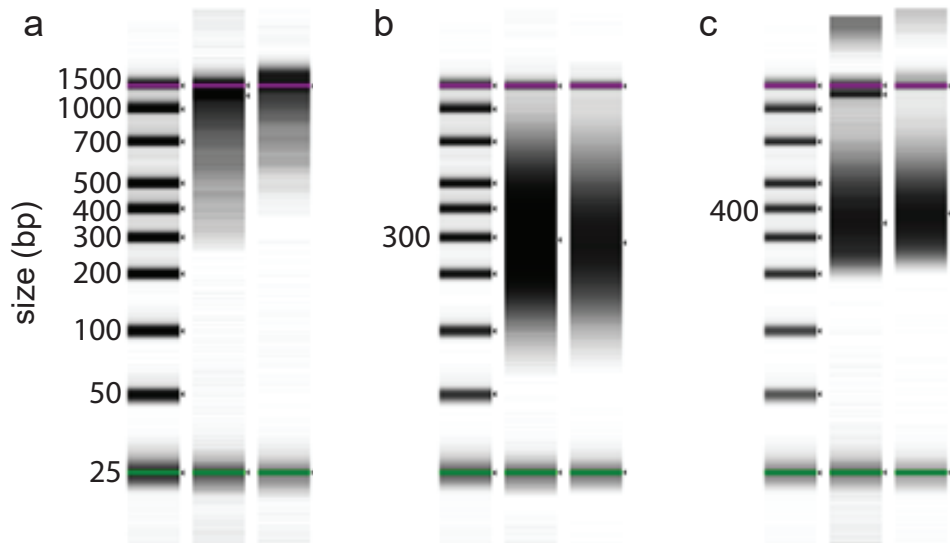

Supplement: Supplementary file 3 — Figure S2. DNA size distributions during TIPseq. a. An Agilent TapeStation image of two samples of purified vectorette PCR DNA is shown with amplicons averaging 1-3 kb. The protocol does not require running samples on TapeStation after vectorette PCR, but this image is included to illustrate the size range. b. The second TapeStation image shows the samples after DNA shearing. The average size distribution for the sheared DNA should be approximately 300 bp. c. As a final quality control, samples should be run on the TapeStation after library prep is completed. This image shows the increase in size from the Illumina adapters and an average library size around 400 bp. (PDF 952 kb) [file 13100_2019_148_MOESM3_ESM.pdf]
