# Supplementary material for: Transposon insertion profiling by sequencing (TIPseq) for mapping LINE-1 insertions in the human genome
Source: Mob DNA. 2019 Mar 8;10:8. doi: 10.1186/s13100-019-0148-5 (PMC6407172; doi:10.1186/s13100-019-0148-5)

Figure S1 (Burns)

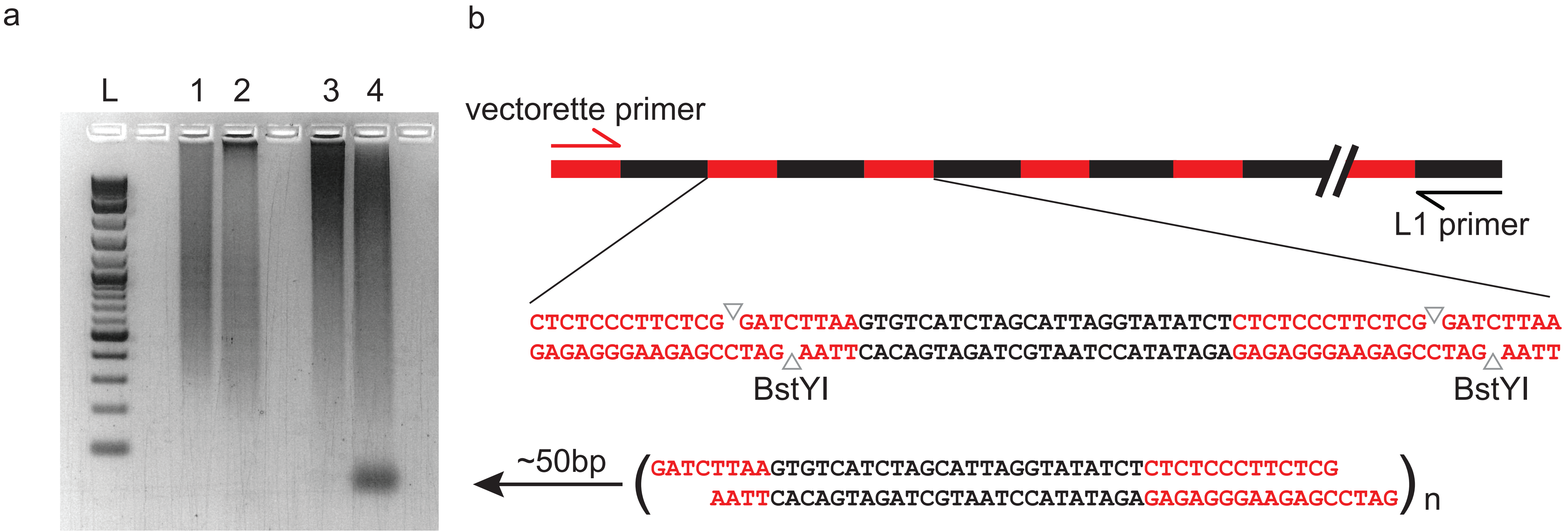

Supplement: Supplementary file 4 — Figure S1. Vectorette PCR amplicons quality control. a. Electrophoresis of vectorette PCR amplicons. The gel image shows five lanes: [L] 2-log ladder (NEB) [1], 2 μg purified PCR amplicons [2], 2 μg purified PCR amplicons digested with BstYI [3], 2 μg purified PCR amplicons ‘contaminated’ with concatemers [4], 2 μg purified PCR amplicons digested with BstYI showing concatemer band at ~ 50 bp. A good vectorette PCR will present as a smear of amplicons averaging 1-3 kb (lane 1). A very high molecular weight smear could indicate contamination (lane 3). b. Schematic of possible vectorette-primer concatemer. Digestion of the amplicons with BstYI, which cuts inside the vectorette primer sequence, will produce a visible band at ~ 50 bp if concatemers are in the PCR product. (PDF 2479 kb) [file 13100_2019_148_MOESM4_ESM.pdf]
